# Detection of Neovascularisation in the Vitreoretinal Interface Slab Using Widefield Swept-Source Optical Coherence Tomography Angiography in Diabetic Retinopathy

**DOI:** 10.1136/bjophthalmol-2020-317983

**Published:** 2020-12-21

**Authors:** Edward S. Lu, Ying Cui, Rongrong Le, Ying Zhu, Jay C. Wang, Inês Laíns, Raviv Katz, Yifan Lu, Rebecca Zeng, Itika Garg, David M. Wu, Dean Eliott, Demetrios G. Vavvas, Deeba Husain, Joan W. Miller, Leo A. Kim, John B. Miller

**Affiliations:** 1 Harvard Retinal Imaging Lab, Boston, MA, USA; 2 Department of Ophthalmology, Harvard Medical School, Boston, MA, USA; 3 Retina Service, Massachusetts Eye and Ear, Department of Ophthalmology, Harvard Medical School, Boston, MA, USA; 4 Guangdong Eye Institute, Department of Ophthalmology, Guangdong Provincial People’s Hospital, Guangdong Academy of Medical Sciences, Guangzhou, China; 5 Wenzhou Medical University Affiliated Eye Hospital, Wenzhou Medical University, Wenzhou, Zhejiang, China; 6 Eye Center of Xiangya Hospital, Central South University, Changsha, Hunan, China

**Keywords:** neovascularisation, vitreoretinal interface slab, diabetic retinopathy, swept-source optical coherence tomography angiography

## Abstract

**Background/Aims:**

To compare the efficacy of diabetic retinal neovascularisation (NV) detection using the widefield swept-source optical coherence tomography angiography (WF SS-OCTA) vitreoretinal interface (VRI) Angio slab and SS-OCT VRI Structure slab.

**Methods:**

A prospective, observational study was performed at Massachusetts Eye and Ear from January 2019 to June 2020. Proliferative diabetic retinopathy (PDR), non-proliferative diabetic retinopathy (NPDR) and diabetic patients with no diabetic retinopathy were included. All patients were imaged with WF SS-OCTA using the 12- x 12-mm Angio scan protocol centred on the fovea and optic disc. The en-face SS-OCTA VRI Angio slab and SS-OCT VRI Structure slab were evaluated for the presence or absence of NV. SS-OCTA B-scan was used to classify NV according to cross-sectional morphology (forward, tabletop, or flat). All statistical analyses were performed using SPSS V.26.0.

**Results:**

One hundred and forty-two eyes of 89 participants were included in the study. VRI Angio detected NV at higher rates compared to VRI Structure (*P* < .05). Combining VRI Angio and Structure improved detection rates compared to VRI Angio alone (*P* < .05). Due to segmentation errors of the ILM, NV with flat morphological classification had lower rates of detection on VRI Angio compared to NV with forward and tabletop morphology (*P* < .05).

**Conclusions:**

WF SS-OCTA 12- x 12-mm VRI Angio and SS-OCT VRI Structure imaging centred on the fovea and optic disc detected NV with high sensitivity and low false positives. The VRI slab may be useful to diagnose and monitor PDR in clinical practice.

## Synopsis/Precis

This study assessed the efficacy of retinal neovascularisation detection using the WF SS-OCTA 12- x 12-mm Angio scan protocol centred on the fovea and optic disc. Combining VRI Angio and Structure improved neovascularisation detection rates.

## INTRODUCTION

Diabetic retinopathy (DR) is the leading cause of vision loss in the working-age population worldwide with increasing incidence and prevalence.[1] Vision-related functional burden is greatest in individuals with proliferative DR (PDR) characterized by ischemia-induced neovascularisation (NV).[2] Early detection of NV is critical to halt disease progression with anti-vascular endothelial growth factor (anti-VEGF) intravitreal injections or laser photocoagulation therapy in advanced disease.[3,4] Traditionally, fluorescein angiography (FA) has been utilized for PDR monitoring by detecting NV-associated dye leakage. However, FA is invasive, time-consuming, and provides only two-dimensional information about retinal vessels.[5]

Optical coherence tomography angiography (OCTA) is a fast and non-invasive imaging modality that generates high-resolution, volumetric images of chorioretinal vasculature.[6] OCTA can detect vascular changes in DR such as vessel density and foveal avascular zone that have been shown to be associated with disease severity and visual function.[7-9] In PDR, OCTA can be used to identify potential biomarkers of active NV.[10] Classification of NV based on origin, en-face features and cross-sectional morphology have been proposed to better understand NV progression and response to therapy.[11,12] However, conventional OCTA is limited by a relatively small field of view compared to FA, which may limit detection of peripheral NV.[6]

Widefield swept-source OCTA (WF SS-OCTA) uses faster scanning speeds to generate images with increased field of view (FOV) in patients with DR.[13] Recently, several groups have demonstrated efficacy detecting NV using the SS-OCTA vitreoretinal interface (VRI) slab, which captures extraretinal lesions extending into the vitreous.[14-16] However, these studies evaluated Montage Angio composite imaging (combining two 15- x 9-mm images or five 12- x 12-mm images) that have potential imaging artifacts, associated segmentation errors and longer acquisition time, thus presenting barriers to widespread adoption in routine clinical practice.[17]

We previously found that the combination of two 12- x 12-mm Angio scans centred on the fovea and on the optic disc detected DR lesions at rates comparable to 15- x 9-mm Montage imaging and thus may be the optimal scan protocol for clinical use.[18] In this study, we assess the efficacy of diabetic retinal NV detection using the WF SS-OCTA 12- x 12-mm en-face VRI Angio slab and SS-OCT VRI Structure slab centred on the fovea and on the optic disc and investigate the association between morphological classification and segmentation errors.

## METHODS

### Subjects

This prospective, observational study was conducted at Massachusetts Eye and Ear from January 2019 to June 2020. Eyes with PDR, NPDR and no DR with imaging using the 12- x 12-mm Angio scan protocol centred on the fovea and optic disc were included. Exclusion criteria were eyes with severe media opacities, signal strength index less than seven using the default settings of the instrument, poor image quality due to severe artifacts, and eyes with concomitant chorioretinal disease. This study was approved by the institutional review board of Massachusetts Eye and Ear, and informed consent was obtained from all subjects. All procedures adhered to the tenets of the Declaration of Helsinki and Health Insurance Portability and Accountability Act regulations.

### Image Acquisition

All participants underwent a full ophthalmic examination and were imaged with a 100 kHz SS-OCTA device (Plex Elite 9000, Carl Zeiss Meditec, Dublin, CA, USA) that uses a laser at a central wavelength at 1060 nm with a bandwidth of 100 nm. Angio (12- x 12-mm) imaging centred on the fovea and optic disc was performed for each eye. Retinal layers were segmented automatically using the built-in custom segmentation of the instrument. The default settings for the VRI slab were utilized, defined as the region 10 to 300 μm above the internal limiting membrane (ILM). The default settings for the whole retina layer were used, defined as the region 70 μm above Bruch’s membrane to the ILM.

### Image Analysis

**Figure 1.**
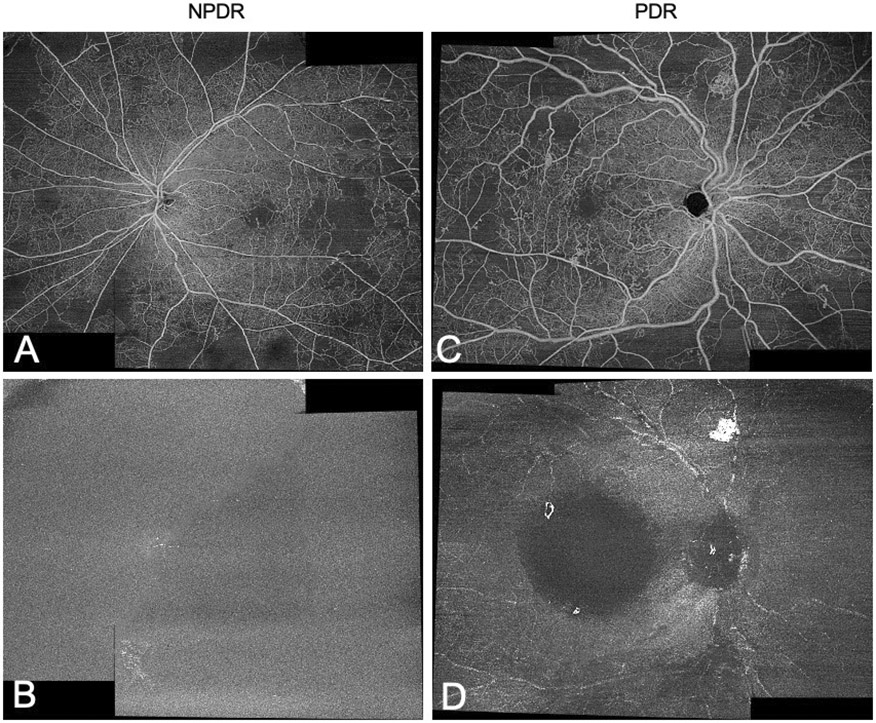
Detection of NV using WF SS-OCTA 12- x 12-mm VRI Angio slab centred on the fovea and on the optic disc. Two 12- x 12-mm images (combined here for demonstration) were evaluated separately. Representative en-face whole retina (A) and VRI Angio (B) images of a NPDR eye. In the PDR eye, NV observed on whole retina imaging (C) are appreciated as corresponding bright signals on VRI Angio (D).

**Figure 2.**
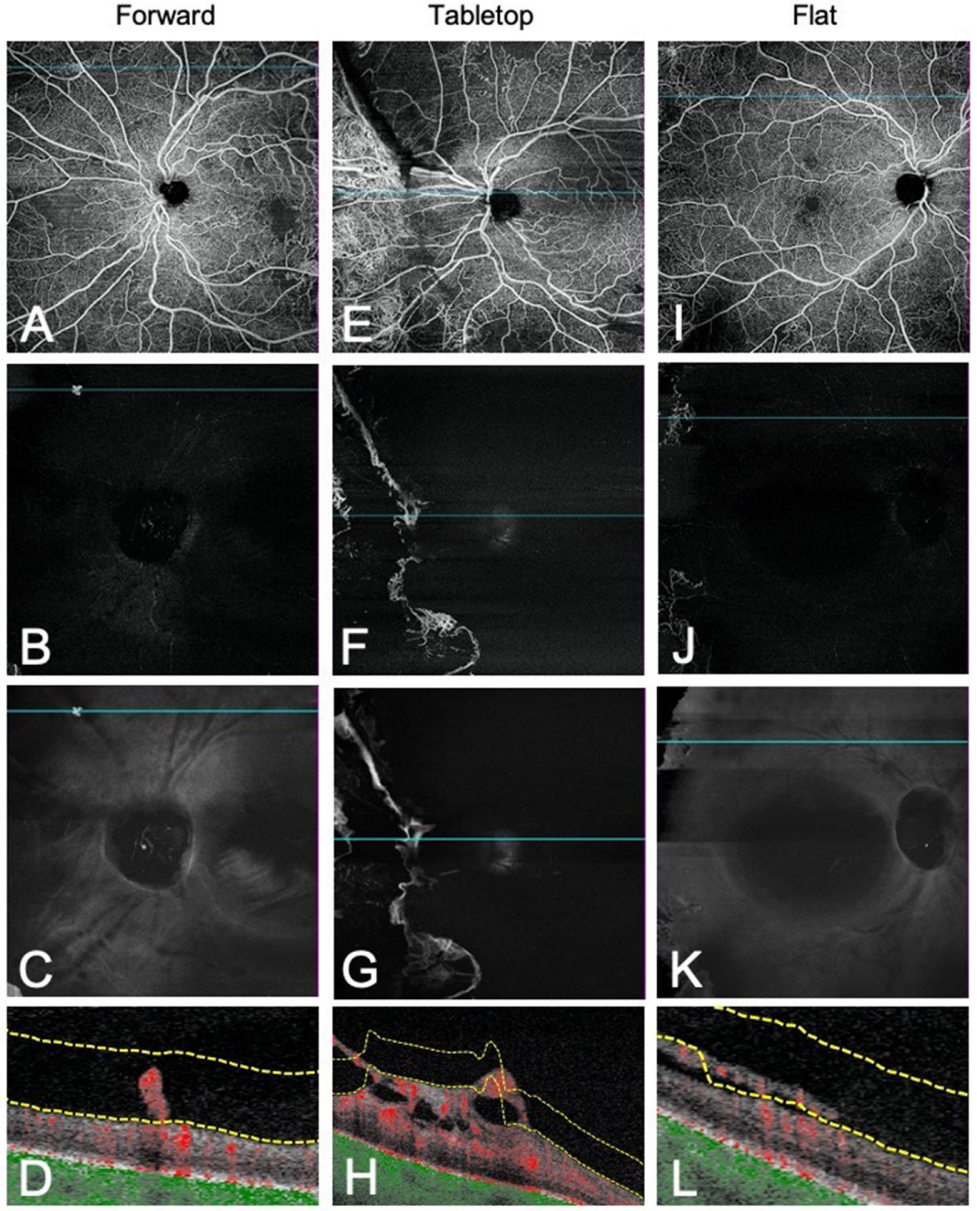
Morphological classification of NV on WF SS-OCTA B-scan. Representative en-face whole retina images, VRI Angio and VRI Structure slabs with co-registered B-scans demonstrating forward (A-D), tabletop (E-H), and flat (I-L) morphologies. Forward NV traversed the posterior hyaloid face and extended into the vitreous. Tabletop NV were displaced anteriorly by vitreous traction but adhered to the retina by vascular “pegs.” Flat NV were confined to the posterior hyaloid face.[12]

SS-OCTA images were independently evaluated by 2 graders (E.L. and Y.C.). For each scan, the en-face 12- x 12-mm SS-OCTA VRI Angio slab and the SS-OCT VRI Structure slab centred on the fovea and optic disc were qualitatively evaluated for the presence or absence of signals, defined as white or grey areas in contrast with the dark background (figure 1). Each signal was subsequently assessed on co-registered SS-OCTA B-scan with blood flow overlay and SS-OCTA en-face whole retina imaging and identified as NV or other retinal lesions (false positives). NV was defined as extraretinal hyperreflective lesions contiguous within a single B-scan or range of B-scans. For each NV, location (NVE, NVD), morphological classification (flat, forward, tabletop), and presence or absence of segmentation errors were determined. Neovascularisation of the disc (NVD) was defined as NV located in the optic disc or within one disc diameter from the margin, and neovascularisation elsewhere (NVE) was defined as NV located outside this area. Morphological classification previously described by Vaz-Pereira et al. was used.[12] Flat NV were localized to the posterior hyaloid face, forward NV traversed the posterior hyaloid face into the vitreous, and tabletop NV were displaced anteriorly by vitreous traction but adhered to the retina by vascular “pegs” (figure 2). NV with both flat and forward components were considered forward NV, and NV with forward and tabletop features were considered tabletop NV. Segmentation errors were defined as incorrect automated segmentation of the ILM resulting in absent signal on VRI. B-scan was used as the reference standard to identify all NV.

### Statistical Analysis

Statistical analyses were performed using SPSS V26.0 (IBM Corporation). Normally distributed continuous variables were presented as means ± standard deviations. Differences between rates of NV detection among different groups were compared using McNemar’s test. Fisher’s exact test was used to compare detection rates by morphological classification. A 2-tailed *P* value of less than 0.05 was considered statistically significant.

## RESULTS

### Demographics

A total of 142 eyes (66 PDR, 60 NPDR, 16 no DR) of 89 participants with type 1 or type 2 diabetes was included in the study. The average age of the participants was 56 ± 14.1 years old; the average duration of diabetes was 19.4 ± 10.5 years; and the average HbA1c level was 8.55 ± 1.89%. A total of 73 participants had diabetic macular oedema, and 71 eyes were treatment naïve at the time of imaging. The mean best-corrected visual acuity was 0.23 ± 0.35 logarithm of the minimum angle of resolution.

### Detection of Neovascularisation Using VRI

A total of 234 NV was identified using SS-OCTA B-scan as the reference standard. PDR eyes accounted for 92.7% (217/234) of NV. The remaining 17 NV were detected in eyes without a clinical diagnosis of PDR, including 15 NV identified in the NPDR group, and 2 NV detected in the no DR group.

VRI Angio detected NV with 91.9% sensitivity, which was higher than the detection rate on VRI Structure (78.6%; *P* < .05) (table 1). Combining VRI Angio and Structure improved the detection rate compared to VRI Angio alone (99.1% vs. 91.9%, respectively; *P* < .05). Subgroup analysis of NVE and NVD showed 90.0% and 100% detection rates on VRI Angio, respectively, which improved to 98.9% NVE detection when combining VRI Angio and VRI Structure (*P* < .05).

### Morphological Classification and Segmentation Errors

**Figure 3.**
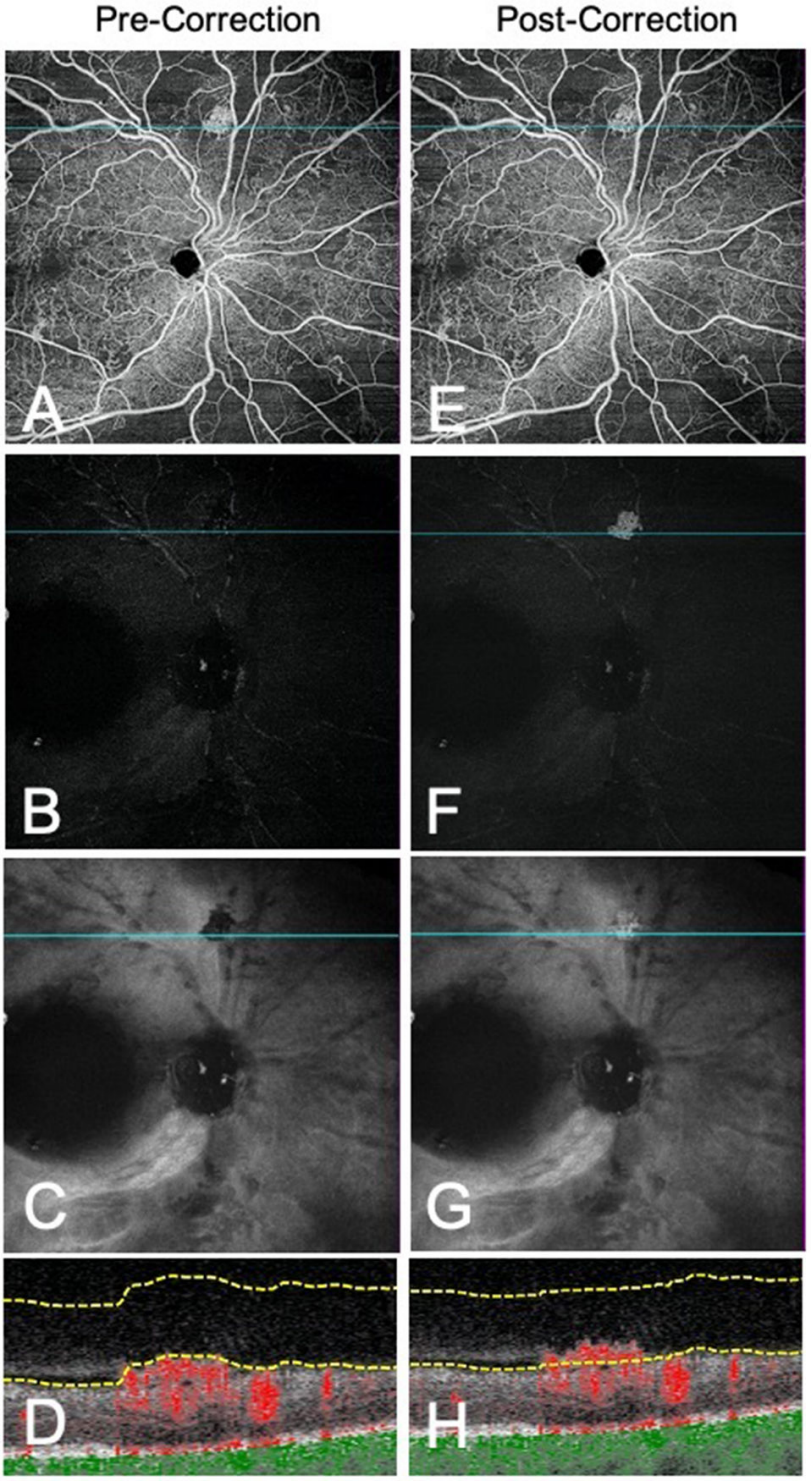
Segmentation error limits detection of flat NV on VRI Angio slab. Representative en-face whole retina images, VRI Angio and VRI Structure slabs with co-registered B-scans before (A-D) and after (E-H) manual segmentation. After manual correction of the ILM, the flat NV is clearly visualized on VRI Angio and VRI Structure slabs. Before manual segmentation, the VRI Structure slab demonstrates a dark inverted signal corresponding to the NV.

A total of 53 flat NV, 138 forward NV, and 43 tabletop NV was confirmed on B-scan. Due to segmentation errors of the ILM, NV with flat morphology had lower rates of detection on VRI Angio compared to NV with forward and tabletop morphology (67.9% vs. 98.6%, 67.9% vs. 100%, *P* < .05) (figure 3). Notably, SS-OCT VRI Structure showed a dark inverted signal corresponding to NV undetected on VRI Angio. Thus, combining VRI Angio and Structure improved detection of flat NV (67.9% vs. 96.2%, *P* < .05).

All 17 flat NV undetected on VRI Angio were associated with automated segmentation errors. Manual correction of the ILM allowed for visualization of flat NV on VRI Angio and VRI Structure. While a subset of flat and tabletop NV demonstrated partial areas of incorrect ILM segmentation, signal was still present on VRI Angio.

### Longitudinal Evaluation of Morphology

Longitudinal analysis of NV morphology included 13 eyes with NV on follow-up imaging. The average follow-up time was 104 ± 60 days. Four PDR eyes had multiple follow-up visits for a total of 18 visits. Of the 18 follow-up eyes, 55.6% (10/18) of eyes had no treatment since the last visit, while 44.4% (8/18) of eyes had treatment including panretinal photocoagulation (4 eyes), anti-VEGF injection (2 eyes), dexamethasone injection (1 eye), and anti-VEGF therapy plus laser retinopexy for a retinal tear (1 eye).

Thirty-seven NV from 18 follow-up eyes were analysed. A total of 9 flat, 20 forward, and 8 tabletop NV was confirmed on B-scan. Five new NV developed in the follow-up period and were not identified on the baseline images. Of the NV identified on prior imaging, 100% (32/32) exhibited the same morphological configuration at follow-up. Thus, NV with and without treatment during the follow-up period did not demonstrate changes in morphological classification. One flat NV was not detected on VRI due to segmentation errors at both the baseline visit and follow-up.

### Distinguishing Neovascularisation from Other Retinal Lesions

**Figure 4.**
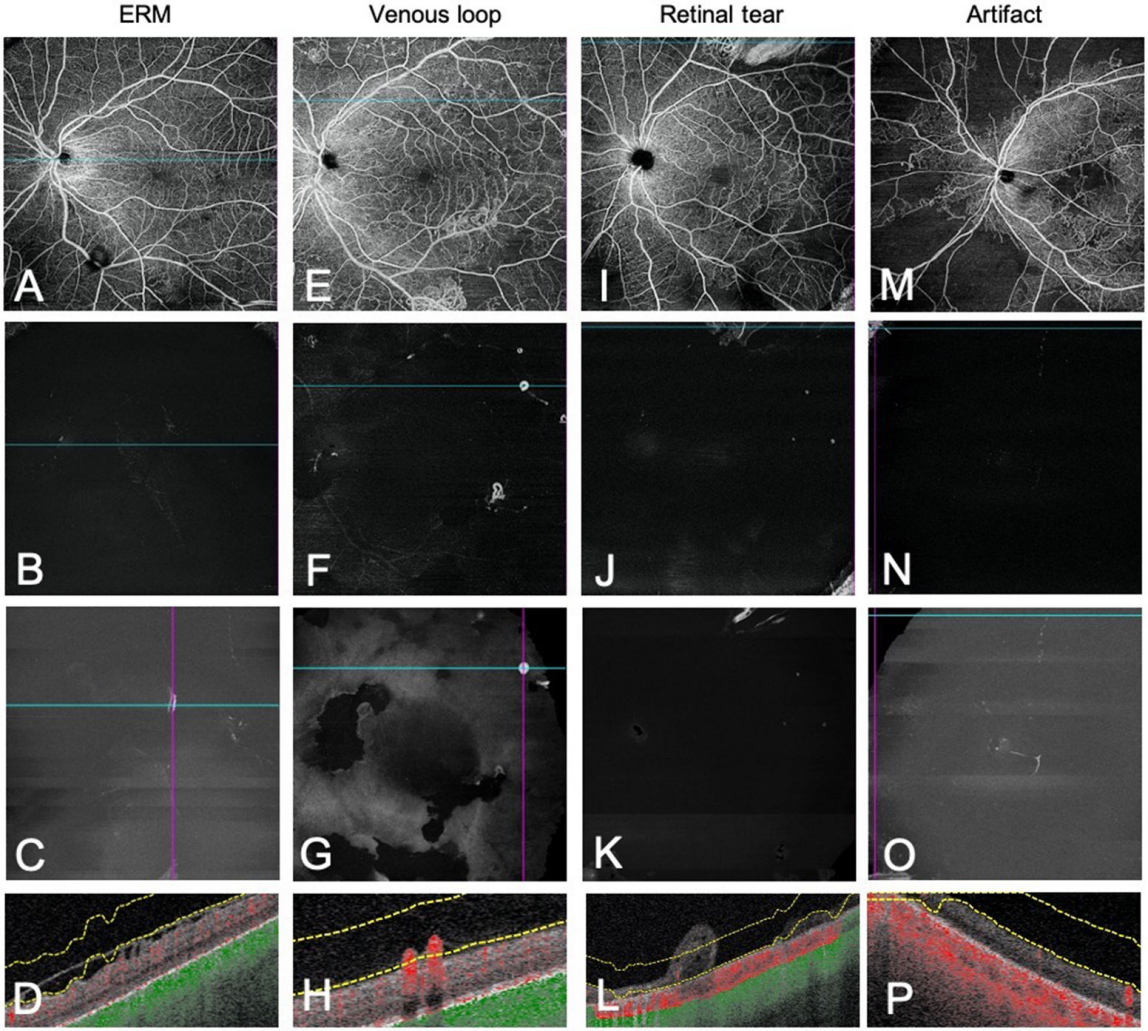
Differentiating NV from other retinal lesions. Representative en-face whole retina images, VRI Angio and VRI Structure slabs with co-registered B-scans highlighting lesions and artifacts that may mimic neovascularisation on VRI. ERMs (A-D) showed no flow on B-scan and did not penetrate the ILM. Venous loops (E-H) resembled forward NV on B-scan but had a distinct omega morphology on en-face whole retina imaging. Retinal tears (I-L) demonstrated detachment of the neurosensory retina on B-scan. Small peripheral artifacts (M-P) due to segmentation error were detected on VRI.

Other lesions appeared similar to NV on VRI and warranted further evaluation on B-scan or en-face whole retina imaging for accurate differentiation (figure 4). Of the 263 total signals detected on VRI Angio, 82.5% (217/263) of VRI signals corresponded to NV, while 17.5% (46/263) of VRI signals were due to other retinal lesions, including 37 epiretinal membranes (ERMs), 7 venous loops, and 2 retinal tears. In addition, 62 peripheral imaging artifacts were detected on VRI.

ERMs (figure 4, A-D) demonstrated no flow or extension into the vitreous on B-scan, distinguishing them from flat NV. Furthermore, ERMs were associated with segmentation errors of the ILM leading to VRI signal. Venous loops (figure 4, E-H) defined as a localized looping trajectory of a vein deviating from its normal linear course, demonstrated signal on VRI and flow on B-scan, resembling forward NV.[19] En-face whole retina imaging highlighted the non-linear omega morphology characteristic of looping vessels and, more fundamentally, identified the feeder vessel to be a vein, thus differentiating venous loops from NV.[20] Retinal tears (figure 4, I-L) mimicked NV fronds on VRI, necessitating assessment of B-scan showing detachment of the neurosensory retina. En-face SS-OCTA images such as the whole retina layer and superficial layer were also used to differentiate retinal tears. Furthermore, vitreous cells seen on B-scan appeared as a diffuse background signal on VRI in contrast with the bright, discrete NV signal.

Sixty-two peripheral imaging artifacts (figure 4, M-P) due to segmentation errors were detected, accounting for 19.1% (62/325) of all VRI signals and 57.4% (62/108) of false positive signals. B-scan showing incorrect ILM segmentation at the edge of the image confirmed the presence of peripheral artifacts detected on VRI.

## DISCUSSION

To our knowledge, this is the first study investigating the utility of WF SS-OCTA 12- x 12-mm VRI Angio and SS-OCT VRI Structure imaging centred on the fovea and on the optic disc for the detection of NV in diabetic retinopathy. In a large cohort of eyes with PDR, NPDR and no DR, the VRI Angio slab detected NV with 91.9% sensitivity. Combining VRI Angio and VRI Structure images improved the detection rate to 99.1%. In a busy clinic, both VRI images can easily be displayed side by side, which may help clinicians screen for PDR based on the presence of NV and monitor PDR progression based on number or size of NV. NV with flat morphology were most likely to be undetected on VRI due to segmentation errors, and longitudinal evaluation of NV in treated and untreated eyes demonstrated no change in morphological classification. Taken together, 12- x 12-mm VRI imaging may be an efficient and non-invasive tool to diagnose and monitor PDR.

While FA is currently the gold standard for evaluating the retinal vasculature, its invasive nature makes the test unsuitable for frequent use at every clinic visit for the screening and monitoring of PDR.[5] SS-OCTA is non-invasive and can be performed routinely for all patients, including those with adverse reactions to FA. Anti-VEGF injections, shown to be non-inferior to panretinal photocoagulation (PRP) for the treatment of PDR in Protocol S, are widely used as a safe and effective treatment for PDR.[21] SS-OCTA can be harnessed to better characterize diabetic retinal NV changes, assess treatment response, and allow for improved medical decision making regarding the optimal treatment schedule for anti-VEGF therapy.

In this study, the 12- x 12-mm Angio imaging centred on the fovea and optic disc were evaluated based on previous work finding this 2-scan protocol to optimize speed and efficacy for detecting DR lesions.[18] Other studies investigating the utility of VRI reported NV detection rates ranging from 73% to 100% using different scan protocols and reference standards for NV detection.[14,16] Using 15- x 15-mm Montage imaging and FA as the gold standard for NV detection, Hirano et al. reported a 73% NV detection rate that improved to 84% with manual segmentation.[14] Pichi et al. reported a 100% detection rate with manual segmentation using the 5-image composite 12- x 12-mm Montage and OCT B-scan as the reference standard. Montage imaging has the advantage of increased FOV at the cost of longer acquisition time requiring increased patient cooperation, as well as lower resolution leading to potential artifacts and segmentation errors.[17] Thus, Montage imaging may not be ideally suited for clinical practice.

SS-OCTA VRI imaging was analysed to determine NV detection efficacy. VRI Angio detected both NVD and NVE at high rates. Although all NVD had detectable signal on VRI Angio, NVD were more likely to have partial segmentation errors of the ILM compared to NVE, which may be explained by the non-linear contour of the optic disc or tendency of NVD to invade through the posterior wall.[22] Notably, 15 NV were identified in eyes with a clinical diagnosis of NPDR, as well as 2 NV detected in diabetic eyes without a DR diagnosis. This finding supports a prior study identifying a subset of small NV with minimal leakage on FA that was detected only on SS-OCTA.[14] Thus, SS-OCTA may be used to detect NV in eyes without evidence of NV on FA, providing a more sensitive tool for PDR diagnosis and earlier initiation of therapy.

In addition, the relationship between NV cross-sectional morphology on SS-OCTA B-scan and segmentation errors was evaluated. NV with the flat configuration had a significantly lower rate of detection compared to forward and tabletop NV. This result is consistent with previous work that found flat NV to be identified on FA but not SS-OCTA due to segmentation errors of the ILM.[14] Manual correction improved visualization of NV but was time-consuming.[23] Deep learning techniques applied to DR screening and diagnosis may be useful for detection of flat NV not adequately captured with automated segmentation, avoiding the need for manual segmentation.[24,25] Forward and tabletop NV had higher detection rates on VRI likely due to greater extension into the vitreous. Interestingly, a subset of flat NV undetectable on SS-OCTA VRI Angio were seen on SS-OCT VRI Structure as dark inverted signals, indicating that the VRI Structure slab may be a useful adjunct to improve NV detection.

Morphological classification of NV was unchanged at follow-up with or without interval laser or anti-VEGF therapy, suggesting that NV morphology may remain constant as size changes, at least in the short term. SS-OCTA has demonstrated progression and regression of NV after PRP as well as the potential for intraretinal microvascular abnormalities to evolve into NV.[26,27] However, changes in morphology are less well characterized. En-face morphological changes such as exuberant vascular proliferation have been shown to be a potential biomarker of active NV in en-face OCTA images.[10] Longer follow-up time is needed to elucidate the dynamic potential of NV using the established morphological classification systems.[11,12]

While this study focused on enhancing NV detection using VRI and the differential detection rates by morphology, further work is needed to investigate the clinical significance of the different NV morphologies, such as the relationship between morphology type and the development of severe complications such as vitreous haemorrhage, tractional retinal detachment, or neovascular glaucoma. In addition, the relationship between morphology and other features, such as hyaloid status, NV location and size, may provide a more nuanced risk assessment of patients with PDR based on NV features.

False positive signals on VRI necessitated further assessment to distinguish NV from other retinal lesions. Evaluation of SS-OCTA B-scan with superimposed flow and en-face whole retina imaging were sufficient to differentiate NV from ERMs, venous loops and retinal tears. Improved visualization of ERMs using VRI may be a useful tool for preoperative surgical planning.[28] In addition, VRI detection of omega retinal venous looping may identify associated nonperfusion areas and predict progression from NPDR to PDR.[29] Peripheral artifacts due to segmentation errors and poor image quality were common. Thus, while VRI may be used to efficiently rule out NV, a multi-step approach including evaluation of co-registered B-scan and en-face whole retina imaging may be necessary to accurately confirm the presence of NV.

This study has some limitations. First, this study qualitatively assessed signals on VRI slabs, which may not capture more subtle morphological features of NV. We also did not differentiate active from inactive NV. However, regardless of activity, NV morphology and associated presence or absence of segmentation errors are likely to be more critical to VRI detection. Finally, we evaluated a relatively small cohort of eyes with longitudinal imaging, although the investigational nature of the device limits its deployment in existing literature. As WF SS-OCTA technology becomes more widely available, a larger sample size with increased follow-up duration will help assess long-term changes in NV morphology.

In conclusion, WF SS-OCTA 12- x 12-mm VRI imaging centred on the fovea and on the optic disc demonstrated efficacy in detecting NV and may be useful for earlier diagnosis and treatment of PDR. Combining VRI Angio with SS-OCT VRI Structure scans improved the detection of all NV morphologies, including the flat type that is more likely to be undetected due to segmentation errors. VRI may serve as a useful tool to diagnose and monitor PDR in clinical practice.

## Figures and Tables

**Table 1.** NV detection rates on VRI Angio, VRI Structure and combined VRI Angio and Structure.

NV Location	VRI Angio	VRI Structure	VRI Combined	*P* Value, Angio vs. Structure	*P* Value, Angio vs. Combined
**NVE**	**90.0%** (171/190)	**74.2%** (141/190)	**98.9%** (188/190)	**<.001** *	**<.001** *
**NVD**	**100%** (44/44)	**97.7%** (43/44)	**100%** (44/44)	**1.000**	**1.000**
**All NV**	**91.9%** (215/234)	**78.6%** (184/234)	**99.1%** (232/234)	**<.001** *	**<.001** *

* The difference was statistically significant (p<0.05)

NV, neovascularisation; NVD, neovascularisation of the disc; NVE, neovascularisation elsewhere; VRI, vitreoretinal interface.
